# ZFAND5 Is an Independent Prognostic Biomarker of Perihilar Cholangiocarcinoma

**DOI:** 10.3389/fonc.2022.955670

**Published:** 2022-07-13

**Authors:** Pei Liu, Yijia Wang, Lingling Duan

**Affiliations:** ^1^ Department of Plastic Surgery, Qilu Hospital Affiliated to Shandong University, Jinan, China; ^2^ Department of Health Care, Central Hospital Affiliated to Shandong First Medical University, Jinan, China

**Keywords:** ZFAND5, prognosis, biomarker, perihilar cholangiocarcinoma, cohort

## Abstract

**Background:**

Cholangiocarcinoma (CCA) is a highly aggressive malignancy with extremely poor prognosis. Perihilar CCA (pCCA) is the most common subtype of CCA, but its biomarker study is much more lagged behind other subtypes. ZFAND5 protein can interact with ubiquitinated proteins and promote protein degradation. However, the function of ZFAND5 in cancer progression is rarely investigated, and the role of ZFAND5 in pCCA is never yielded.

**Materials and Methods:**

In this study, we established a pCCA cohort consisting of 72 patients. The expression of ZFAND5 in pCCAs, and the paired liver tissues, intrahepatic bile duct tissues and common bile ducts (CBD) tissues were detected with IHC. ZFAND5 mRNA in pCCAs and CBDs was detected with qRT-PCR. The pCCA cohort was divided into ZFAND5^low^ and ZFAND5^high^ subsets according to the IHC score. The correlations between ZFAND5 expression and clinicopathological parameters were assessed bychi-square test. The prognostic significance of ZFAND5 expression and clinicopathological parameters was estimated by univariate analysis with Kaplan-Meier method, and by multivariate analysis with Cox-regression model.

**Results:**

Expression of ZFAND5 in pCCAs was substantially higher than that in interlobular bile ducts and common bile ducts, but lower than that in liver tissues. The ZFAND5^low^ and ZFAND5^high^ subsets accounted for 44.4% and 55.6% of all pCCAs respectively. ZFAND5 ^high^ patients had much lower survival rates than the ZFAND5^low^ patients, with the average survival time as 31.2 months and 19.5 months respectively. ZFAND5 was identified as an independent unfavorable prognostic biomarker of pCCA with multivariate analysis.

**Conclusion:**

ZFAND5 expression was up-regulated in pCCAs compared with the CBDs. We identified ZFAND5 as an independent biomarker of pCCA, which could provide more evidence for the molecular classification of pCCA, and help stratify the high-risk patients based on the molecular features.

## Introduction

Cholangiocarcinoma (CCA) is a highly aggressive malignancy originating from bile duct (1). CCA has several clinical manifestations such as dormant symptoms, severe malignancy, rapid progression, early metastasis, easier invasion and recurrence (2). These clinical features attribute to the poor prognosis of CCA. The 5-year survival rate ranging from 5% to 20% (3, 4). Base on anatomical locations, CCA can be classified as three subtypes, which are the intrahepatic CCA (iCCA), perihilar CCA (pCCA), and distal CCA (dCCA) (5). pCCA has the highest prevalence among CCAs, taking up about 50% of all CCAs. pCCA is not sensitive to systemic chemotherapy and patients always suffer early recurrence (6). Till 2020, the first targeted drug of CCA, pemigtinib, just emerged for treatment of patients with FGFR2 fusion, which only accounted for 10%-15% iCCA and 5% iCCA/dCCA (7). The treatment options of CCA are very dismal, and pCCA is the most less studied CCA subtype in the field of biomarker and target therapy (8). Therefore, identifying new biomarkers and therapies to expand the pCCA treatment options and improve life span of pCCA is very important.

ZFAND5 (AN1-type zinc finger protein 5, also named ZNF216) is a 23-kD cytosolic protein with one A20 zinc finger domain and one AN1-type zinc finger domain (9). It is a member of the zinc finger AN1-type domain family, which are comprised of 8 members (10). Among the AN1-type domain family, ZFAND2a and ZFAND5 are featured by their ability to increase the overall protein degradation (11). ZFAND5 interacts with poly-ubiquitinated proteins with the N-terminal A20-type zinc finger domain (12). ZFAND5 is highly expressed in the brain and skeletal muscle, and is detectable in many tissues, and is reported to participate in muscle atrophy and osteoclasts differentiation (13). However, the expression of ZFAND5 in many other tissues are not clear.

The function of ZFAND5 in cancer progression is rarely investigated. Previous study suggested ZFAND5 as a biomarker of hepatocellular carcinoma indicating favorable prognosis (14). However, other studies showed that ZFAND5 was highly expressed in more aggressive nasopharyngeal carcinoma cells (15), and ZFAND5 may be associated with proliferation of colon cancer (16), indicating that ZFAND5 may have pro-tumor effects. Taken together, the current knowledge of ZFAND5 role in tumor progression is very poor and controversial. Here we investigated the expression of ZFAND5 in pCCA with immunohistochemistry (IHC), and estimated the clinical significance of ZFAND5 detection by investigating the correlation between ZFAND5 and clinicopathological parameters. Moreover, we estimated the prognostic value and the independent prognostic significance of ZFAND5 in pCCA.

## Materials and Methods

### Patients Cohort

The retrospective cohort was comprised of 122 patients who underwent the radical surgery of pCCA from 2017.1 to 2021.6 in Qilu Hospital of Shandong University and the Central Hospital of Shandong First Medical University, which constituted the initial cohort. 72 patients were enrolled from the initial cohort into the validation cohort if they (1) had available specimens for IHC detection and available follow-ups, (2) survived the perioperative period, (3)had no other malignancies. Moreover, another consecutive perspective cohort comprised of 10 patients was established if the patients underwent radical surgery and had enough pCCA tissues and adjacent CBD tissues for quantified real-time PCR (qRT-PCR). The pCCAs were staged as the 8^th^ American Joint Committee on Cancer/Union for International Cancer Control (AJCC/UICC) Tumor-Node-Metastasis (TNM) classification system.

All specimens were obtained with informed consent from patients. All experiments were approved and supervised by the Ethics Committee of Qilu Hospital of Shandong University and the Central Hospital of Shandong First Medical University.

### Tissue Microarray Construction

The slides were stained with hematoxylin and eosin staining for double confirmation of diagnosis and selection for proper tumor area to make TMA. The tumor area for TMA construction was determined by a senior pathologist. The core biopsies with 1.5 mm in diameter were taken from the selected area and arranged into TMA slides.

### qRT-PCR

In the perspective cohort, total mRNA was purified with TRIzol reagent (Thermo Fisher, Waltham, MA, USA). The extracted total RNAs were reversely transcribed into cDNA with SuperScript Reverse Transcriptase (Thermo Fisher). The quantified real-time PCR was performed with QuantiFast Probe RT-PCR Plus Kit (Qiagen, Germany). mRNA level of ZFAND5 was standardized with GAPDH as an internal control by the 2^-ΔΔCt^ method. All primers were designed as follows:

ZFAND5: forward primer 5’-GCTAGTGGTTCCAACAGTCCT-3’,

reverse primer 5’-TCGGGGTAGTTATTTTGTCCTCT-3’;

GAPDH: forward primer 5’-TGTGGGCATCAATGGATTTGG-3’,

reverse primer 5’-ACACCATGTATTCCGGGTCAAT-3’.

### IHC Staining

The streptavidin-peroxidase IHC was used to detect the ZFAND5 expression in pCCA tissues. In brief, the TMA was deparaffinized and rehydrated with xylene and graded alcohol, and then immersed in 3% hydrogen peroxide to inactivate the endogenous peroxidase. After that, the TMA slides were incubated in citrate buffer (pH = 6.0) and boiled using a microwave for optimal antigen retrieval. The unspecific antigen binding was blocked by bovine serum albumin incubation. TMA slides were incubated in the primary antibody of ZFAND5(NBP1-80609, Novus Biologicals, Minneapolis, MN, USA) at dilution as 1:100 overnight at 4°C, and then rinsed by phosphate buffered saline (PBS) for 3 times. After that, TMA was incubated in the secondary antibody labeled with biotin (Beyotime, Beijing, China) for 1 hour at room temperature. The antigen was finally visualized by peroxidase reaction with 3,3-diaminobenzidine (DAB) solution (Beyotime).

### IHC Score Evaluation

The IHC results were semi-quantified by IHC scores which were determined by the staining intensity as well as the positive cell percentage. The staining intensity was defined as negative (score 0), weak (score 1), moderate (score 2), or strong (score 3). The positive cell percentage was calculated as <25% (score 1), 25%-50% (score 2), 50%-75% (score 3) and >75% (score 4). The final IHC scores were the products of the score (staining intensity) multiplied by the score (positive cell percentage), varying from 0 to 12. The IHC scores were further divided into the ZFAND5^low^ and ZFAND5^high^ scores based on the cut-off in the receiver operating characteristic (ROC) curve, which was set as the cases with the highest specificity plus sensitivity. In our study, the cut-off was 3.5, representing that scores ≥4 were set as high ZFAND5.

### Statistical Analysis

All the statistical analyses were performed with software SPSS 22.0. The correlations between ZFAND5 and clinicopathological parameters were estimated by the chi-square test. The overall survival curves were plotted with Kaplan-Meier method and the statistical difference between different subsets were compared with the log-rank test. The Cox proportional hazard regression model was used to assess the independent prognostic significance of ZFAND5 and clinicopathological parameters. The statistical significance between IHC scores of different subtypes was analyzed with one-way ANOVA, and the statistical significance of qRT-PCR results was assessed with paired t test. P value less than 0.05 was considered as statistically significant.

## Results

### ZFAND5 is Highly Expressed in pCCA

**Figure 1:**
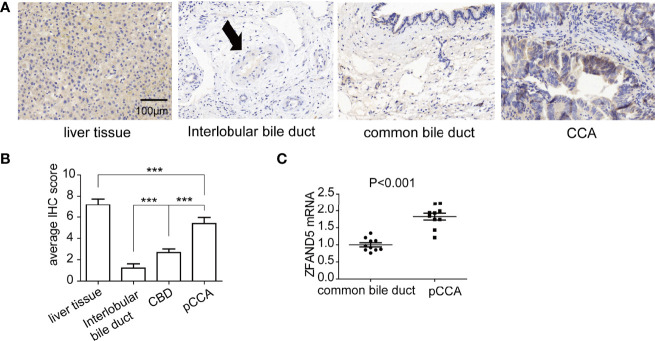
The expressions of ZFAND5 in pCCAs, liver tissues, interlobar bile ducts tissues, and common bile ducts tissues. **(A)** The expressions of ZFAND5 in pCCAs, liver tissues, interlobar bile ducts tissues, common bile ducts tissues were detected by IHC. Scale bar: 100 μm. The arrow indicates the interlobar bile duct. **(B)** The expressions of ZFAND5 in pCCAs, liver tissues, interlobar bile ducts tissues, and common bile ducts tissues were estimated by the IHC scores. *** represents P < with one-way ANOVA test. **(C)** mRNAs of ZFAND5 in pCCAs and common bile ducts were detected with qRT-PCR. The statistical significance was analyzed by paired t test.

Firstly, we established a cohort comprised of 72 pCCA patients. There were 31 male patients and 41 female patients, with an average age as 56.5 years old (**Table 1**). The expression of ZFAND5 in the 72 pCCAs, and their paired liver tissues, intrahepatic bile ducts tissues and common bile ducts (CBD) tissues were detected with IHC (**Figure 1A**). In these tissues, ZFAND5 was abundantly expressed in cell cytoplasm. Moreover, The IHC scores of pCCA were substantially higher than those of interlobular bile ducts tissues and CBD tissues, but lower than those of hepatic tissues(**Figure 1B**).

**Table 1:** The basic infromation of the pCCA cohort.

Clinicopathological parameters		Number	Percentage
	
Age (years)	<60	42	58.33%
	≥60	30	41.67%
Sex	Male	31	43.06%
	Female	41	56.94%
Tumor size (cm)	<3cm	36	50.00%
	≥3cm	36	50.00%
Histograde	I	10	13.89%
	II	54	75.00%
	III	8	11.11%
T stage	T2	48	66.67%
	T3	20	27.78%
	T4	4	5.5.6
N stage	N0	51	70.83%
	N1	21	29.17%
TNM stage	II	34	47.22%
	IIIA	15	20.83%
	IIIB	19	26.39%
	IVA	4	5.56%
ZFAND5	Low	32	44.44%
	High	40	55.56%

FAND5, AN1-type zinc finger protein 5; pCCA, perihilar cholangiocarcinoma.

Moreover, we established a perspective cohort including 10 consecutive pCCA patients, and detected the ZFAND5 mRNA of pCCAs and adjacent CBD tissues. As expected, the ZFAND5 mRNAs in pCCAs were extensively higher than those in CBDs, indicating a potential role of ZFAND5 in tumorigenesis and progression of pCCA(**Figure 1C**).

### The Correlations Between ZFAND5 Expression and Clinicopathological Parameters

**Figure 2:**
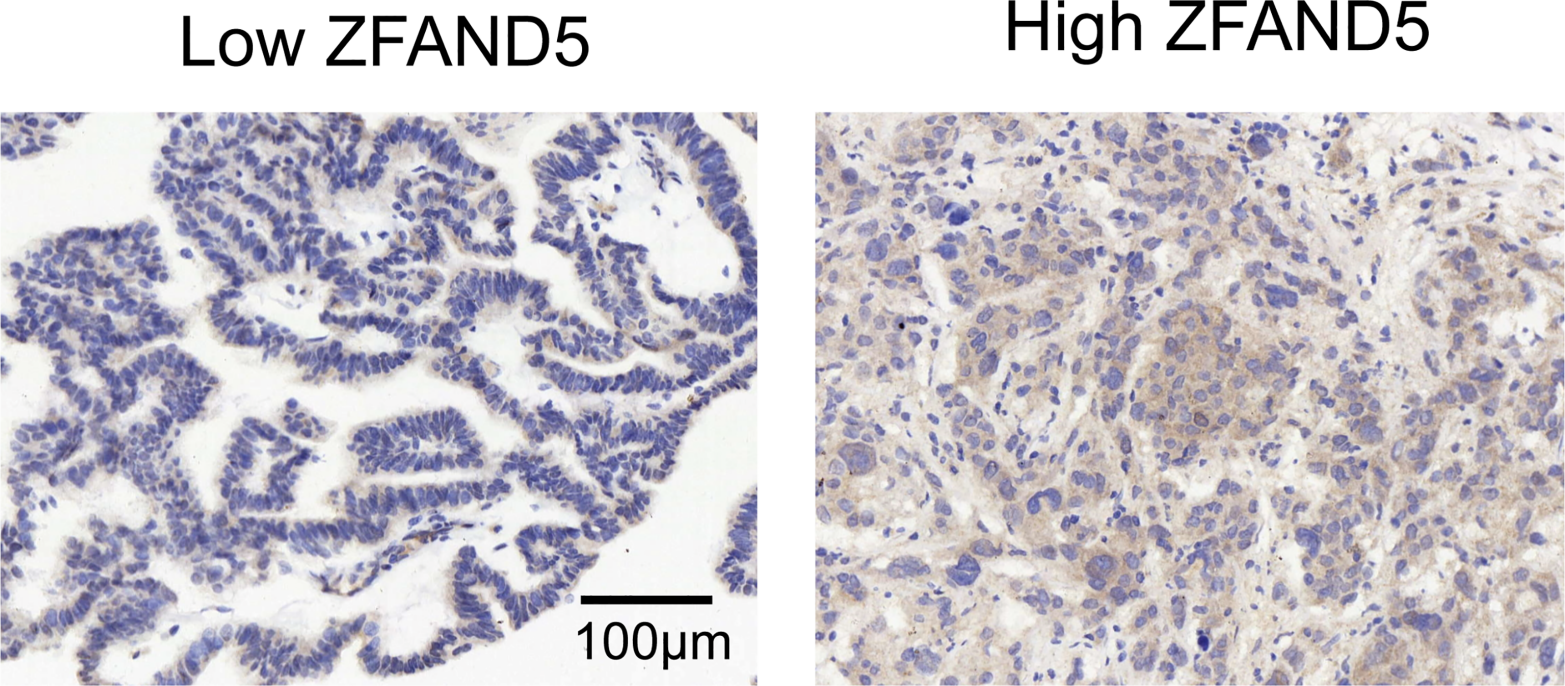
The cohort was divided into ZFAND5low and ZFAND5high subsets. The representative images of low and high ZFAND5 expression. Scale bar: 100 μm.

We divided the pCCA cohort into subsets with low or high expression of ZFAND5 based on the IHC score (**Figure 2**). There were 32 ZFAND5^low^ patients and 40 ZFAND5^high^ patients, accounting for 44.4% and 55.6% of all pCCAs respectively (**Table 1**). The correlations between ZFAND5 expression and clinicopathological parameters were further assessed with chi-square test. The enrolled clinicopathological parameters included the gender, age of patients, tumor size, histological grade, T stage, N stage and TNM stage. M stage was not included because all patients were present without metastasis. No significant correlation between ZFAND5 and these parameters was observed. The only potential ZFAND5-associated factor was tumor size. High expression of ZFAND5 appeared to be correlated with larger tumor size, though the statistical significance was not remarkable(P=0.155) (**Table 2**).

**Table 2:** The correlation between ZFAND5 and clinicopathological parameters.

Clinicopathological parameters		ZFAND5		
		Low	High	P*
Age (years)	<60	18	24	0.748
	≥60	14	16	
Sex	Male	15	16	0.558
	Female	17	24	
Tumor size (cm)	<3cm	19	17	0.155
	≥3cm	13	23	
Histograde	I	5	5	0.663
	II	27	35	
T stage	T2	22	26	0.737
	T3+4	10	14	
N stage	N0	22	29	0.797
	N1	10	11	
TNM stage	II	14	20	0.598
	III+IV	18	20	

ZFAND5, AN1-type zinc finger protein 5. *calculated by chi-square test.

### ZFAND5 Was a Prognostic Biomarker of pCCA

**Figure 3:**
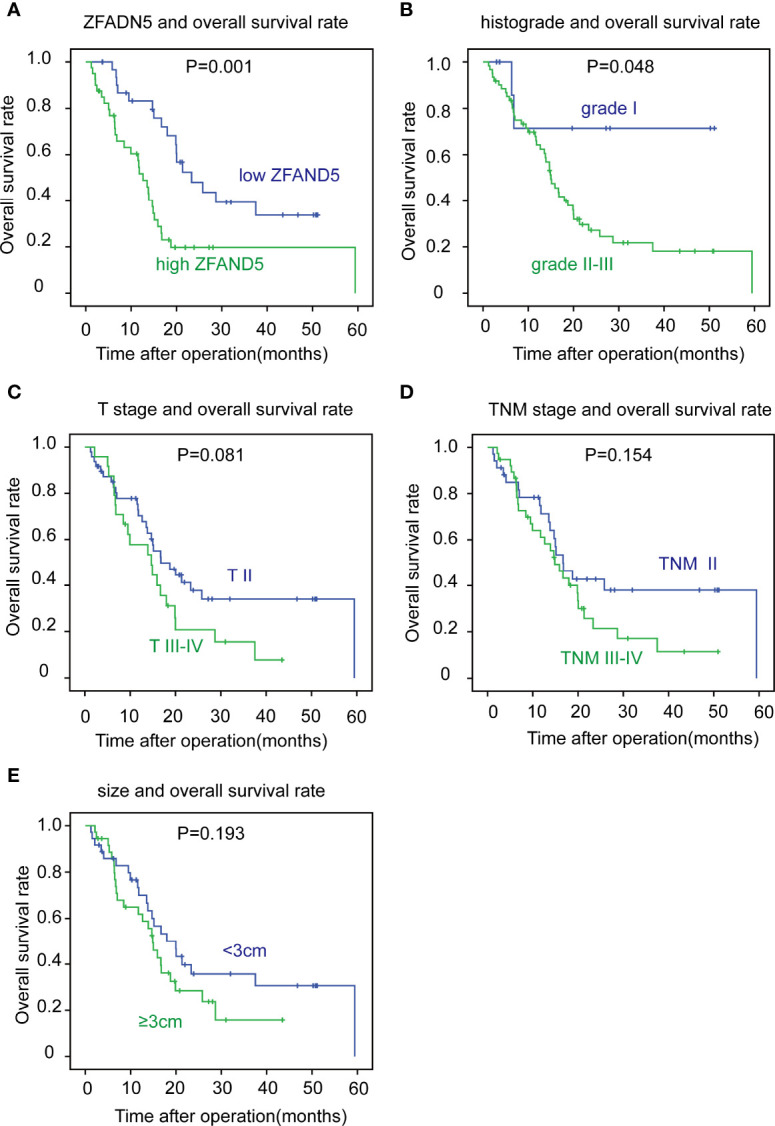
The correlation between clinicopathological factors and overall survival rates. The patients were divided into different subsets according to ZFAND5 expression **(A)**, histological grade **(B)**, T stage **(C)**, TNM stage **(D)** and tumor size **(E)**. The survival curves were plotted by Kaplan-Meier method, and the statistical significance was analyzed with the log-rank test.

With Kaplan-Meier methods, we plotted the survival curves of all clinical parameters and ZFAND5 (**Table 3**). Noticeably, high expression of ZFAND5 correlated with lower overall survival rates of pCCA, indicating that ZFAND5 was an effective prognostic biomarker of pCCA (**Figure 3A**). The average survival time of ZFAND5^low^ and ZFAND5^high^ patients were 31.2 months and 19.5 months, with the 5-year overall survival rates as 31.9% and 0% respectively. In addition to ZFAND5, the advanced histological grade could also predict a poor prognosis of pCCA (**Figure 3B**). Advanced T stage, TNM stage and tumor size tended to correlate with low survival rates, with a noteless significance (**Figure 3C–E**). Other parameters did not exhibit strong correlations with the overall survival rates.

**Table 3:** The prognostic significance in univariate analysis.

Clinicopathological parameters		average OS time	5-year OS	P*
Sex	Male	18.6	9.1	0.377
	Female	27.5	0	
Age (years)	<60	27.1	0	0.367
	≥60	20.3	16.5	
Tumor size (cm)	<3cm	28.1	0	0.193
	≥3cm	18.1	15.8	
Histograde	I	28.4	71.4	0.048
	II+III	22.1	0	
T stage	T2	28.5	0	0.081
	T3+4	16.8	13.5	
N stage	N0	26.8	0	0.368
	N1	19.7	8.3	
TNM stage	II	29.9	0	0.154
	III+IV	18.9	11.4	
ZFAND5	Low	31.2	31.9	0.001
	High	19.5	0	

ZFAND5, AN1-type zinc finger protein 5; pCCA, perihilar cholangiocarcinoma. *log-rank test.

### The Independent Prognostic Risks of pCCA

Moreover, we enrolled all these clinicopathological parameters into the Cox-regression hazard model to identify the independent prognostic factors. In this multivariate analysis, ZFAND5 was identified as an independent prognostic biomarker of pCCA. The odds of ZFAND5^high^ patients was 4.33 fold times higher than that of ZFAND5^low^ patients to suffer from pCCA-related death (**Table 4**). Additionally, high histological grade was also an independent factor indicating the unfavorable outcome (*P*=0.039, HR=5.18).

**Table 4:** The independent prognostic factors were determined by multivariate analysis.

Clinicopathological parameters		pCCA		
		HR	95%CI	P*
Sex	Male	1		
	Female	0.62	0.33-1.17	0.142
Age (years)	<60	1		
	≥60	1.46	0.77-2.74	0.243
Tumor size (cm)	<3cm	1		
	≥3cm	1.17	0.55-2.50	0.698
Histograde	I	1		
	II+III	5.18	1.08-24.8	0.039
T stage	T2	1		
	T3+4	1.31	0.63-2.75	0.469
N stage	N0	1		
	N1	1.92	0.98-3.75	0.057
ZFAND5	Low	1		
	High	4.33	2.0-9.2	<0.001

HR, hazard ratio; CI, confidence interval; ZFAND5, AN1-type zinc finger protein 5; pCCA, perihilar cholangiocarcinoma. *Cox-regression hazard model.

## Discussion

CCA is a highly heterogeneous and complicated malignancy. The three subtypes of CCA, iCCA, pCCA and dCCA, have distinct clinical manifestations, treatment options and outcomes (6). However, their biological characters are not as more distinguished as their clinical features. In recent years, many molecular fingerprints of CCA have been identified, defining different molecular CCA patterns (1, 17). However, most studies on molecular CCA patterns are focused on iCCA, because iCCA specimens are much easier to get, compared with pCCA. pCCA and dCCA were separated as distinct subtypes in 2007 according to the 7^th^ AJCC/UICC (18). But the understanding of the molecular differences between pCCA and dCCA is extremely poor. More studies on the molecular characters of CCA, especially pCCA, could deepen the understanding of differences among CCA subtypes, and help discover prognostic biomarkers.

Even in the field of CCA biology, the studies of pCCA are much fewer compared with iCCA and dCCA. Although pCCA has substantially higher incidence, the radical surgery of pCCA is more difficult than iCCA and dCCA. Hepatectomy and pancreaticoduodenectomy are the radical surgical approaches for iCCA and dCCA respectively, but the radical surgery of pCCA is extensively lower compared with iCCA and dCCA (19). The anatomical complexity of hepatic portal determines that pCCA is much easier to invade the hepatic artery and portal vein, making the radical surgery extremely difficult, which further results in that the specimen obtainment of pCCA is difficult, and that large pCCA cohort study is rare. Our cohort consisting 72 pCCA patients is a relatively large-sample-sized cohort. Our results demonstrated that ZFAND5 was a highly effective biomarker indicating the unfavorable prognosis of pCCA (*P*=0.001). Our study could provide more evidence for the molecular classification of pCCA, and help stratify the high-risk patients based on the molecular features.

Cancer cells have many hallmarks in cell metabolism and energy utilization (20). For example, in shortage of available nutrients, cancer cells can exert adaptive metabolic pathways to maintain their viability by the self-catabolic processes such as autophagy. Protein ubiquitination and degradation are the key segments in the self-catabolic processes, and the molecular characters of proteasome mechanisms and regulation need further studies. ZFAND5 interacts with both Ub and proteasomes, and it may function as a linker between substrates and 26S proteasome. Previous study showed that ZFAND5 could increase multiple proteasomes activities and extensively induce proteolysis by the Ub-proteasome pathway (12). However, the exact mechanism of ZFAND5-induced protein ubiquitination is still unknown. For example, whether ZFAND5 interacts with the Ub-binding substrates, and whether ZFAND5 functions as a E3 ligase is still unknown. We speculated that the high expression of ZFAND5 may be attributed to the enhanced autophagy of CCA, which is a promising topic but requires further experiments.

Till now, the studies of ZFAND5 physiological function are very insufficient, and the reports on the oncogenic role of ZFAND5 are extremely poor. In addition to an activator of proteasome, other functions of ZFAND5 were also reported. Previous study demonstrated that ZFAND5 could bind and stabilize mRNAs with AU-rich elements in 3’-untranslated regions (21). In addition, ZFAND family are mainly stress-associated proteins. For example, expression of ZFAND2a (also named AIRAP) is increased in cases that misfolded proteins are accumulated, such as arsenite exposure, heat shock, or proteasome inhibition (22). ZFAND2a could promote proteins degradation by binding to proteasomes and thus increased their peptidase activity (21, 22). The exact mechanism of ZFAND5 up-regulation in pCCA, and the function of ZFAND5 in pCCA progression is worthy of further investigation.

The role of ZFAND5 in tumor immunity is not investigated. On one hand, ZFAND5 can be induced by cytokines including RANKL, TNFα, and IL-1β, on the other hand, ZFAND5 can inhibit the activation of transcription by NF-κB, TNFα, or IL-1β (13, 23). However, the exact mechanism of how ZFAND5 influences the cytokine and how it participates in inflammation is still unknown. Given that ZFAND5 affects cytokine expression and is involved in immunology, it would be a very interesting topic to further study the role of ZFAND5 in tumor response to immune checkpoint inhibitors.

In summary, here we established a pCCA cohort consisting of 72 patients, and identified ZFAND5 as an independent biomarker of pCCA. The odds of ZFAND5^high^ patients was 4.33 fold times higher than that of ZFAND5^low^ patients to suffer from pCCA-related death. Our results provide more evidence for the molecular classification of pCCA, and help stratify the high-risk patients based on the molecular features.

## Data Availability Statement

The original contributions presented in the study are included in the article/supplementary material. Further inquiries can be directed to the corresponding author.

## Ethics Statement

All experiments were approved and supervised by the Ethics Committee of Qilu Hospital of Shandong University and the Central Hospital of Shandong First Medical University. The patients/participants provided their written informed consent to participate in this study.

## Author Contributions

PL and YW performed the experiments, PL and LD designed the study, collected the specimens and wrote the paper. All authors contributed to the article and approved the submitted version.

## Conflict of Interest

The authors declare that the research was conducted in the absence of any commercial or financial relationships that could be construed as a potential conflict of interest.

## Publisher’s Note

All claims expressed in this article are solely those of the authors and do not necessarily represent those of their affiliated organizations, or those of the publisher, the editors and the reviewers. Any product that may be evaluated in this article, or claim that may be made by its manufacturer, is not guaranteed or endorsed by the publisher.
